# Molecular Basis of Obesity: Current Status and Future Prospects

**DOI:** 10.2174/138920211795677921

**Published:** 2011-05

**Authors:** Hélène Choquet, David Meyre

**Affiliations:** 1 Ernest Gallo Clinic and Research Center, Department of Neurology, University of California, San Francisco, Emeryville, CA 94608, USA; 2 Departments of Clinical Epidemiology and Biostatistics, McMaster University, Hamilton, ON L8N 3Z5, Canada

**Keywords:** Candidate gene, childhood obesity, copy number variation, gwas, heritability, linkage, monogenic, polygenic.

## Abstract

Obesity is a global health problem that is gradually affecting each continent of the world. Obesity is a heterogeneous disorder, and the biological causes of obesity are complex. The rapid increase in obesity prevalence during the past few decades is due to major societal changes (sedentary lifestyle, over-nutrition) but who becomes obese at the individual level is determined to a great extent by genetic susceptibility. In this review, we evidence that obesity is a strongly heritable disorder, and provide an update on the molecular basis of obesity. To date, nine loci have been involved in Mendelian forms of obesity and 58 loci contribute to polygenic obesity, and rare and common structural variants have been reliably associated with obesity. Most of the obesity genes remain to be discovered, but promising technologies, methodologies and the use of “deep phenotyping” lead to optimism to chip away at the ‘missing heritability’ of obesity in the near future. In the longer term, the genetic dissection of obesity will help to characterize disease mechanisms, provide new targets for drug design, and lead to an early diagnosis, treatment, and prevention of obesity.

## INTRODUCTION

Since 1980, the mean body mass index (BMI) worldwide increased by 0.4-0.5 kg/m² per decade in adults 20 years and older with 502 million adults worldwide classified as obese (BMI ≥ 30 kg/m²) by 2008 [1]. The worldwide prevalence of childhood overweight and obesity have increased from 4.2% in 1990 to 6.7% in 2010 and this trend is expected to reach 9.1% in 2020 [2]. This global health problem is gradually affecting each continent of the world. 

High rates of weight gain during infancy may increase a person’s later risk of obesity. There are growing numbers of obese children developing diseases formerly considered to be an “adult” condition, such as type 2 diabetes, nonalcoholic fatty liver disease, sleep apnea and hypertension [3]. Obesity-associated diseases are now believed to lead to a shortened lifespan [4]. As the incidence of obesity-related diseases increase among adults as well as children, the consequences of the obesity epidemic on the economy of developed countries should be considered as a major priority [5]. 

The environmental causes of childhood obesity involve an unhealthy diet and physical activity patterns as well as early-life factors such as diabetes exposure *in utero*, larger size for gestational age, shorter breastfeeding duration and more rapid infant weight gain [6]. However, obesity is the result not only of several environmental risk factors, but also genetic predisposition [7]. Understanding the many causal factors leading to the complex trait of obesity may help develop more targeted and effective therapies.

## PROGRESS IN DEFINING THE MOLECULAR BASIS OF OBESITY

### Obesity is a Heritable Disorder

Family history of obesity is a well-established risk predictor for obesity in childhood. The risk in a child is 2.5-4-fold higher if one of their parents is obese and 10-fold higher if both parents are obese, compared to having both parents of normal weight [8]. The familial risk for obesity (the risk ratio to be obese for an individual if a first degree relative is obese compared with individuals in the population who have only normal-weight first degree relatives) is comprised between 1.5 and 5 depending on the severity of obesity [9]. The familial aggregation of individual’s body size is not of a recent concept: Sir Francis Galton mentioned this observation in his book “Natural Inheritance” in 1889 [10]. However, as familial resemblance can be explained by shared environments as well as genetic factors, the specific influence of genes in determining early-onset obesity was investigated 35 years ago with the emergence of twin and family studies.

In 1977, Feinleib and colleagues [11] studied the correlations for weight in 250 monozygotic (100% of genome shared) and 264 dizygotic (50% of genome shared) male veteran twin pairs and established for the first time that familial aggregation for obesity results mainly from genetic influence. These results were confirmed in more than 4,000 monozygotic and dizygotic twin pairs in 1986. Strong heritability values for body mass index (BMI) were observed for the same subjects at 20 years (h²= 0.77) and at 45 years (h²=0.84). Heritability represents the proportion of phenotypic variation in a population that is attributable to genetic variation among individuals [7]. Adoption studies have strengthened the evidence of a strong genetic influence on human body weight. Body corpulence of adopted children was shown to strongly correlate with BMI of their biologic parents, whereas no correlation was observed with the BMI of their adoptive parents [12]. Furthermore, correlation values for BMI of 0.66-0.7 were estimated by Stunkard *et al*. [13] for monozygotic twins reared apart, 0.66-0.74 for monozygotic twins reared together, 0.15-0.25 for dizygotic twins reared apart and 0.27-0.33 for dizygotic twins reared together suggesting that genetic influences on body-mass index are substantial, whereas the family environment alone has minor influence. Many twin and family studies including children or adolescents have been published since these pioneering observations, providing heritability values for BMI between 0.20 and 0.86 [14-17]. 

Longitudinal studies have demonstrated that heritability estimates tend to increase from infancy to childhood [18], from childhood to pre-adolescence [15] and from pre-adolescence to adolescence [17], mirroring the growing exposure to obesity-prone environments that subjects with genetic propensities tend to favor. Heritability studies have also shown that longitudinal BMI change from adolescence to young adulthood is an heritable trait and that genetic factors modulating BMI levels are only partially shared with those modulating change in BMI across time, supporting a complex etiology of BMI and BMI change [19]. Heritability of obesity as a binary trait, as well as for BMI in all ranges of the population, involved overall equates to the same set of genes [15]. 

Heritability values for BMI / obesity can be modified by specific environmental exposures. A high level of physical activity can substantially reduce the influence of genetic factors on BMI in both young adults and older adults [20, 21]. Even if heritability estimations of BMI are similar in sex-specific analyses, some sets of genes explaining the BMI variation may be different in adolescent as well in adult males and females [14, 22]. Ethnicity may also interact with the genetic predisposition to obesity and ethnic-specific genetic factors are likely to modulate human body weight. The proportion of European genome in admixed African or Indian Native populations is inversely correlated with BMI, supporting the idea that specific gene subsets may account for genetic susceptibility to obesity in different ethnic backgrounds [23, 24]. Surprisingly, the major societal changes that accompanied the transition to an “obesogenic” environment (environments that promote gaining weight) did not have a major impact on the genetic predisposition to obesity. The values of heritability for BMI in childhood remain high (h²=0.77) even in the current “obesogenic” environment [16], and many of the same genes seem to be involved in establishing genetic susceptibility to obesity in the pre- as in the post-obesity epidemic period [25]. Heritability estimates for obesity-related traits are consistent among Nigerian, Jamaican and US black people despite contrasted environmental conditions and different obesity prevalence (5% in Nigeria, 23% in Jamaica and 39% in the USA) [26]. 

Beyond BMI, heritability studies have also been conducted on endophenotypes related to obesity in young individuals. Strong genetic influences have been evidenced for intermediate traits such as percentage of body fat [27], waist circumference [28], eating behaviour [29], level of physical activity [30] or energy expenditure [31].

### Mendelian Forms of Non-Syndromic Obesity 

Monogenic forms of obesity refer to a single gene disorder leading to a highly penetrant form of the disease. The study of extreme human obesity caused by a single gene defects has provided a glimpse into the long-term regulation of body weight. For example, defects in eight genes involved in the neuronal differentiation of the paraventricular nucleus and in the leptin/melanocortin pathway lead to monogenic forms of early-onset severe obesity, demonstrating the critical role of the leptin-melanocortin system critical for energy balance in humans [32]. The eight genes are leptin (*LEP*), leptin receptor (*LEPR*), proopiomelanocortin (*POMC*), prohormone convertase 1 (*PCSK1*), melanocortin 4 receptor (*MC4R*), single-minded homolog 1 (*SIM1*), brain-derived neurotrophic factor (*BDNF*) and its receptor TrkB coded by the neurotrophic tyrosine kinase receptor type 2 gene (*NTRK2*).

#### Recessive Forms of Monogenic Obesity

Recessive forms of obesity caused by homozygous / heterozygous compound loss of function mutations in five genes (*LEP*, *LEPR*, *POMC*, *PCSK1* and *MC4R*) have been reported to date [33-37]. Complete inactivation of these five genes invariably results in severe hyperphagia and fully penetrant form of early-onset extreme obesity in humans. Additional phenotypic features associated with these recessive forms of obesity are more specific: individuals with congenital leptin receptor or leptin deficiency present alterations in immune function and frequent childhood infections of the respiratory tract associated with high rates of premature death. They also manifest delayed puberty due to hypogonadotropic hypogonadism, and hypothyroidism [33, 34]. The clinical features of individuals with congenital leptin receptor deficiency are usually less severe than those with congenital homozygous leptin deficiency, and hypothyroidism is less common [38]. Individuals with complete POMC deficiency present in their early life with hypoadrenalism secondary to ACTH deficiency and develop hypoglycemia, jaundice and neonatal death which is associated with severe liver cholestasis. POMC-deficient individuals of European ancestry have pale skin and red hair [35], but those with congenital POMC deficiency can harbor normal hair and skin pigmentation in other ethnic backgrounds [39]. Individuals with complete *PCSK1 *deficiency harbor reactive hypoglycemia and perturbations in endocrine function including episodes of severe diarrhea which indicate an important role for the PC1/3 enzyme in enteroendocrine cells [36, 40]. Individuals with complete MC4R deficiency harbor an increased lean mass, increased bone mineral density and tall stature [37]. 

The study of recessive forms of monogenic extreme obesity has been useful in delineating the role of genes from the leptin / melanocortin pathway in human physiology but explains a small percentage of obesity in the population. Indeed these recessive forms of obesity are excessively rare and are mostly identified in families with a high level of consanguinity (consanguinity increases the probability to carry two deleterious copies of the same gene). Thus far, 14 individuals with complete LEP deficiency have been identified worldwide, 13 subjects with complete LEPR deficiency, seven with complete POMC deficiency, three with complete PCSK1 deficiency and 20 with complete MC4R deficiency.

#### Partial Gene Deficiency and Obesity Features

Heterozygosity for deleterious coding mutations in *MC4R* [41] or *POMC* [42] has been associated with a non fully penetrant form of obesity, whereas partial LEP or LEPR deficiency has been associated with a higher percentage of body fat mass [38, 43]. On the opposite side, the eight heterozygous carriers of *PCSK1* loss of function mutations reported to date, have been described as clinically unaffected [44]. This result is intriguing, since heterozygous *PC1*^N222D/+ ^mice present with an obesity-intermediary phenotype [45] and heterozygotes PC1-null mice tend to be mildly obese [46]. Even if heterozygous carriers of deleterious mutations in *MC4R*, *POMC*, *LEP* and *LEPR* present a milder and incompletely penetrant form of obesity in comparison with the obligatory severe obesity phenotype caused by homozygous / heterozygous compound loss of function mutations in the same genes, they are likely to contribute for a non-negligible fraction of obesity at the population level. 

MC4R deficiency is the common cause of monogenic obesity. Starting from a *MC4R* loss of function mutation frequency of 0.07% reported in the general populations [47], we can expect 426,701 heterozygous carriers and only 149 homozygous / compound heterozygous carriers in the US population (N=305,000,000). Using an average penetrance of 60% for heterozygous carriers and 100% for homozygous and compound heterozygous carriers, as previously reported in literature [41], partial MC4R deficiency may explain obesity in 256,021 individuals, whereas complete MC4R deficiency may be the cause of obesity for only 149 subjects in the US population.

Haplo-insufficiency for BDNF, TrkB and SIM1 has been associated with severe hyperphagic obesity, accompanied by syndromic features in humans [48-51]. BDNF and its receptor TrkB are involved in proliferation, survival, and differentiation of neurons during development and post-natal synaptic plasticity in the central nervous system, especially in hypothalamic neurons. SIM1, the mammalian homologue of *Drosophila sim,* is a transcription factor playing a major role in neuronal differentiation of the paraventricular nucleus of the hypothalamus, a critical brain region for food intake regulation. No human case of complete deficiency for BDNF, TrkB and SIM1 are reported in literature. This is consistent with the phenotypes observed in mice. Partial deficiency of Bdnf, TrkB or Sim1 in mice induces hyperphagia, obesity and developmental features [52-54], whereas complete deficiency for Bdnf or Sim1 is lethal [52, 53] and complete deficiency for TrkB dramatically reduces life span [55].

#### Genome Structural Variations

In the last few years, the advent of genome-scanning technologies has led to the discovery that genetic differences among people can derive from lost or duplicated segments of chromosomes, called copy number variants (CNVs) or structural variants [56]. CNVs contribute significantly to the genetic architecture of human obesity. Rare deletions in the region p11.2 of the chromosome 16 have been reported in about 0.5-0.7% of individuals with severe obesity in two independent studies [57, 58], and the link between deletions at 16p11.2 and obesity has been confirmed in three follow-up reports [59-61]. The 16p11.2 deletion interval identified in these studies encompasses about 30 genes. The SH2B adapter protein 1 (*SH2B1*) is one of these genes and is an excellent candidate gene to link the 16p11.2 deletion to obesity. Indeed, SH2B1 modulates leptin sensitivity and *Sh2b1* knock-out mice develop hyperphagia and obesity [62]. In addition, the *SH2B1* locus was recently associated with common obesity by genome-wide association studies (GWAS) [63, 64]. 

A recent study identified 17 rare CNV loci previously unreported in the public domain and only found in obese but not in lean children of European ancestry. Eight out of 17 CNVs were also found in obese children of African ancestry, but not in lean control subjects of the same ethnicity [65]. Finally, Wang *et al*. [60] investigated the potential role of large and rare CNVs in moderate and extreme obesity and demonstrated that rare CNVs > 2 Mb were present in 1.3% of obese subjects but were absent in lean controls. Several CNVs disrupt known candidate genes for obesity, such as *NAP1L5*, *UCP1* and *IL15 *[60]. The examination of rare CNVs offers novel insights into the genetic architecture of obesity. 

### Polygenic Forms of Obesity 

The search for gene variants associated with polygenic forms of obesity is based on the common disease-common variant hypothesis. This hypothesis states that multiple common, interacting disease alleles contribute to common diseases (each variant at each gene having a modest effect on the disease phenotype) and are well represented in human populations [66]. Three main approaches have been used to identify novel gene variants associated with polygenic obesity: candidate gene, genome-wide linkage and genome-wide association studies.

#### Candidate Gene Studies

Association studies with candidate genes have been widely used for the study of complex traits but have proven largely unsuccessful. Several hundreds of obesity candidate genes have been selected for genetic association study on the biological, physiological or pharmacological evidence of their role in body weight regulation, but most of the positive association signals initially reported have never been consistently replicated in follow-up replication studies [67]. Large meta-analyses have been reported for only a small number of variants, and so far only two genes have been reliably associated with obesity phenotypes using this approach. 

Targeted disruption of Mc4r results in obesity in mice, loss-of-function coding mutations in *MC4R* lead to the more common form of monogenic human obesity [41, 68] and significant evidence of linkage for obesity-related traits has been reported on chromosome 18, where the *MC4R* gene resides [69]. *MC4R* is therefore a highly relevant candidate gene for common obesity. Two gain-of-function [70] infrequent coding polymorphisms (I251L and V103I) in *MC4R* have been negatively associated with obesity [71, 72]. The 103I variant was associated with a 20% lower risk for obesity in a meta-analysis of 39,879 subjects of European ancestry [72] and the 103I allele carriers had a 31% lower risk for obesity in a meta-analysis of 3,526 individuals from six East Asian studies [73]. The 251L allele was associated with a 50% lower risk for obesity in a meta-analysis of 11,435 European subjects [72]. 

Loss-of-function rare mutations in the *PCSK1* gene have been associated with obesity both in humans and rodents [36, 45]. In addition, four independent genome-wide linkage studies for obesity-related traits delineate a common 5.6-Mb interval on chromosome 5q where *PCSK1* resides [74]. We recently showed that the relatively infrequent (minor allele frequency: 5%) single nucleotide polymorphism (SNP) N221D induces a 10.4% significant reduction of PC1/3 catalytic activity, and is associated with a 34% increased risk for obesity in European populations [74]. The N221D variant was also associated with BMI level in an independent sample of 32,000 European subjects [64]. These two examples indicate that the candidate gene approach can be successful if: 1) stringent criteria are applied to gene and SNP selection process and 2) large scale association studies are performed. 

#### Genome-Wide Linkage Studies

Genome-wide linkage scans involve the genotyping of families recruited for the high recurrence of a disease using highly polymorphic microsatellite markers that are regularly spaced across the whole genome, followed by a calculation of the degree of linkage of the marker to a disease trait. Genome-wide linkage approaches led to the successful identification of > 1,200 genes involved in Mendelian human diseases, however the application of genome-wide linkage in the analysis of complex genetic traits has been more controversial [75]. More than 80 linkage studies for obesity-related traits have been reported, showing significant evidence for linkage (LOD-score > 4) for some of them [76, 77]. However, a meta-analysis of 37 published studies containing data on over 31,000 individuals was unable to confirm a major locus for obesity [78], as observed in meta-analyses for other complex diseases [79]. 

Possible explanations may include genes influencing adiposity are of very small effect, with substantial genetic heterogeneity and variable dependence on environmental factors, but this does not explain why significant peaks of linkage have been reported in individual studies at many different chromosomal locations. A plausible alternative explanation is that rare variants with high disease penetrance may be a common explanation for linkage peaks observed in complex traits like obesity [42, 80]. This is in line with statistical simulations predicting that odds-ratios must be high (OR > 2) to induce significant peaks of linkage in modest family sample sets [75]. Attempts to identify new obesity genes by genome-wide linkage strategies have been mostly unsuccessful, genetic variants in only two genes have shown association with obesity, contribution to the initial linkage peak and have been confirmed in subsequent replication studies. 

The only significant evidence of linkage for childhood obesity was obtained on chromosome 6q22.31-q23.2 in 115 French pedigrees [76]. Restriction of the linkage interval under study by comparison of eight independent linkage studies and subsequent positional candidate gene approach led to the identification of a three-allele risk haplotype (K121Q, IVS20delT-11, A→G +1044TGA; QdelTG) in the ectonucleotide pyrophosphatase / phosphodiesterase 1 (*ENPP1*) gene that showed association with childhood obesity and contribution to the observed linkage with childhood obesity [81]. The *ENPP1* QdelTG haplotype was associated with adult moderate and morbid obesity and type 2 diabetes and was also associated with increased serum levels of soluble ENPP1 protein in children [81]. Interestingly, *ENPP1* gene variation was found to be independently associated with obesity-related traits in Mexican American pedigrees by a positional candidate gene approach [82]. The function of the gene can be directly related to obesity and type 2 diabetes as ENPP1 inhibits insulin receptor signaling [83]. The link between ENPP1 and insulin resistance has been evidenced in genetic mouse models as well [84]. Insulin resistance in brain induces hyperphagia and obesity in mice [85] and insulin resistance is a strong predictor of subsequent development of obesity in children [86]. The association of the *ENPP1* risk haplotype with childhood obesity has been replicated in a German study [87] but further replication is needed to provide an unequivocal confirmation.

In 2002, Stone and colleagues [77] identified a major predisposition locus for sever obesity in White American females on chromosome 4p15-p14. Subsequent positional cloning effort by the same team led to the identification of a non-synonymous polymorphism (R125W) in the *TBC1D1* gene associated with severe familial obesity in women only and accounting for the majority of the evidence of linkage on chromosome 4p15-14 [88]. The association of the *TBC1D1* R125W polymorphism with familial severe obesity in females and linkage at 4p15-p14 was replicated in an independent French cohort [89]. Further replication is now needed at this stage, and the gender-specific association of the R125W variant with obesity remains unexplained. TBC1D1 protein function involves adipogenesis process [90], insulin signalling [91] and lipid use in skeletal muscle [92] and inactivation of Tbc1d1 confers leanness in mice [92]. These observations further support the candidacy of* TBC1D1* as a ‘thrifty’ gene as recently proposed by Koumanov *et al*. [93].

#### Genome-Wide Association Studies

Genome-wide association studies (GWAS) are a relatively new way for scientists to identify genes involved in human diseases and have revolutionized the search for genetic common variants contributing to complex diseases. This method interrogates the genome for several hundred-thousand single nucleotide polymorphisms (SNPs) across the genome, and identifies the SNPs that occur more frequently in people with a particular disease than in those without the disease. As of June 2010, there have been more than 900 published genome-wide associations for 165 traits (http://www.genome.gov). The combinations of three major breakthroughs have made genome-wide comprehensive association studies possible: 1) the emergence of the notion of linkage disequilibrium block [94], the determination of the human genome SNP map through the International Hapmap Consortium [95] (this led to the conclusion that 80% of the common genetic variation (>14 million variants) in subjects of European ancestry can be captured by genotyping 300,000 carefully selected single nucleotide polymorphisms (SNPs) [96]); 2) the development and commercialization of new methods for high throughput genotyping using SNP microarrays [97] and 3) the recruitment of large scale case control and population-based cohorts with both DNA and phenotypes available [98]. 

In 2007, four different approaches led to the identification of variation in the intron 1 of Fat mass and obesity associated (*FTO*) gene as the major contributor to polygenic obesity in populations of European ancestry [99-102]. Frayling *et al*. [99] identified *FTO* through a GWAS for type 2 diabetes in UK subjects, variants in the *FTO* gene showing a strong association with T2D mediated through BMI [99]. Scuteri *et al*. [101] identified *FTO* by using a GWAS approach for BMI in the genetically isolated population of Sardinia. Hinney *et al*. [102] identified *FTO* by using GWAS for early-onset extreme obesity in a German case control study. Dina *et al*. [100] unexpectedly found a strong association between *FTO* and obesity, by performing a population structure approach with a set of 48 SNPs in a French obesity case control design. The 16% of adults of European ancestry who were homozygous for the risk allele weighed about 3 kilograms more and had 1.67-fold increased odds of obesity when compared with those not inheriting a risk allele [99]. Apart from European populations, *FTO* was shown to be the top association signal for BMI (*P*=1 x 10^-7^) in a recent GWAS including 8,842 Korean subjects [103]. 

Four large GWAS meta-analyses in general populations of European descent (16,876 < N < 123,865) confirmed the strong association of the *FTO* locus with BMI and identified 35 additional SNPs in 33 loci robustly (P < 5 x 10^-7^) associated with BMI (Table **1**). Multiple independent association signals were reported at the *FTO*, *MC4R* and *BDNF* loci [63, 104]. Altogether these loci only explained 1.45% of the variance in BMI (0.34% explained by the SNP in intron 1 of *FTO* alone), suggesting that many additional common genetic variants associated with BMI remain to be discovered [104]. Each additional risk allele increases BMI of 0.17 kg/m² and body weight of 435-551 grams in adults of 160-180 cm in height [104]. Interestingly, SNPs that modulate BMI in general adult populations are also associated with BMI in pediatric populations [105], and are associated with an increased risk for early-onset [64, 104] or adult obesity [106]. 

The genetic architecture of extreme childhood and adult obesity has also been investigated using case control GWAS designs [102, 107-109]. Nine loci have been associated with extreme obesity at the genome-wide level, five of them influencing BMI / waist circumference as well as risk for extreme obesity (*FTO, MC4R, TMEM18, MSRA, NPC1*) [102, 104, 107-110], four loci being more specific of genetic risk for extreme obesity (*MAF, PTER, PRL, SDCCAG8*) [107, 109] (Table **1**). Overall, these data indicate that the genetic architecture of BMI and extreme obesity are mostly overlapping in children as well as adults, and that extreme obesity may represent the extreme of the phenotypic spectrum of BMI rather than a distinct condition [108]. Several of the likely causal obesity genes identified through GWAS studies (*FTO, MC4R, POMC, SH2B1, BDNF, NPC1, NRXN3* and* NEGR1*) are highly expressed or known to act in the central nervous system, indicating a key role for central regulation of food intake in obesity susceptibility in line with monogenic forms of human obesity [64, 104]. 

Beyond the study of BMI, four GWAS have investigated the genetic architecture of body fat distribution, estimated by waist circumference (WC) or waist to hip ratio (WHR) measurements [110-113]. Nineteen loci have been identified and five of them only are associated with BMI / obesity (*FTO, MC4R,* *NRXN3, TFAP2B, MSRA*) (Table **1**). However, it is to noted that WC or WHR measurements were adjusted for BMI in the two larger GWAS meta-analyses, to identify SNPs associated with central adiposity and those independently of overall adiposity [110, 113]. The biological role of several likely causal candidate genes identified by GWAS pinpoint a key role of adipose tissue development and function in determining body fat distribution [113]. 

Common copy number variants (CNVs) are in high linkage disequilibrium with SNPs in the human genome suggesting that disease-associated common structural variants can be identified by SNP-based whole-genome association studies [114]. Using a SNP tagging approach, a 45-kb deletion near the *NEGR1* gene and a 21-kb deletion 50 kb upstream of *GPRC5BT* have been recently and convincingly associated with BMI variation [64, 104], and a 2.8-kb common duplication 87 kb from the *LY86* gene has been conclusively associated with WHR [113]. A common CNV on chromosomes 10p11.22 has been associated with BMI in a Chinese population [115] and covers four genes, one of them (*PPYR1*) being a plausible obesity candidate gene [115]. The association of CNV 10p11.22 with obesity has been confirmed in two independent studies [60, 61]. CNVs are plausible functional causal variants due to their potential impact on gene expression [116], and genes located nearby these CNVs might be prioritized for fine mapping and functional follow-up.

## FUTURE PROSPECTS

### New Approaches to Chip Away at the “Missing Heritability” of Obesity

Although there is strong evidence that heritability of human body weight is high, only a small fraction of the variance in BMI can be explained by the current list of genetic factors suggesting potential sources of missing heritability [104]. One hypothesis about missing heritability from GWAS has focused on the possible contribution of variants of low minor allele frequency (MAF), defined as 0.5<MAF<5 %, or of rare variants (MAF<0.5 %). GWA genotyping arrays used in the first waves of GWAS classically included 300,000-500,000 SNPs and they provided only an exhaustive coverage of the common SNP variation (MAF > 5%) in the genome [96], signifying that rare variants associated with complex diseases have not been captured in these studies. Low frequency variants may have much stronger effects on the disease risk than common variants without demonstrating clear Mendelian segregation, and may explain an important part of the missing heritability [117]. Common and rare structural variation, including CNVs, such as insertions and deletions and copy neutral variation, may account for some of the unexplained heritability [118]. Imprinted genes [119], gene x gene and gene x environment interactions [120, 121], ethnic-specific disease loci [103] or disease-associated haplotypes that are not identified by single SNP analyses [122] may also account for a substantial part of the missing heritability of obesity. 

The establishment of many population-based studies that have collected genome-wide data on genetic variation has recently led to the formation of consortia facilitating powerful GWAS meta-analyses for BMI or WHR [104, 113]. Upcoming GWAS meta-analyses for continuous or extreme obesity phenotypes in larger sample sizes are likely to complete the current list of obesity predisposing genes, and will enable gene x environment or gene x gene well-powered GWAS studies. The completion of family-based GWAS for obesity-related traits will be useful to identify disease-associated imprinted loci and haplotypes [118]. The integration of data issued from expression and DNA arrays offers the possibility to identify true disease-associated SNPs that do not reach a genome-wide significant level of association in initial GWAS analysis. This approach recently led to the identification of *F13A1* as a valuable candidate gene for obesity [123], and SNP prioritization programs integrating expression QTL information are now emerging [124]. A new generation of dense DNA arrays (including up to 5 millions SNP and CNVs) based on the detailed information of rare and common single nucleotide and copy number variation in the human genome provided by the 1000 Genome project [125], combined with the development of adequate statistical methodologies [126], will enable the identification of novel rare variants predisposing to obesity. Numerous rare variants may be detected in a gene or region but they may have disparate effects on phenotype. One strategy to study associations between such variants and disease might be pooling rare variants together using logical criteria and analyzing them as a single group [127]. 

Others strategies may shortly lead to a more exhaustive picture of the rare variants explaining highly penetrant forms of obesity. High-resolution homozygosity mapping in large consanguineous pedigrees is a powerful approach to discover novel obesity loci with a recessive mode of inheritance, as recently exemplified in syndromic forms of obesity [128]. Exome capture and parallel sequencing strategies in carefully selected unrelated cases and controls have proven successful for gene identification [129] and this approach should be successfully extended in the future to large pedigrees with a Mendelian pattern of obesity. The high occurrence of Mendelian patterns of inheritance observed in multigenerational pedigrees with extreme obesity, the fact that > 130 genes lead to a severe obesity phenotype in mice when down or up-regulated, and that every single monogenic gene explains a weak fraction of human obesity suggests that many monogenic genes remain to be discovered [77]. In addition to the deletion at 16p11.2, the identification of additional rare structural variants associated with highly penetrant forms of obesity by genome-wide association approaches are likely to be followed by systematic resequencing approaches for genes located in genome structural variation intervals, that may help to identify additional Mendelian obesity genes. Altogether, these novel methodologies are likely to fill the gap of the “missing heritability” of obesity in the near future.

### New Phenotypes for New Genes

#### On the Use of BMI Variable in GWAS

Heritability studies in young and older adults have recently shown that genetic factors modulating BMI levels are only partially shared with those modulating change in BMI across time, supporting a complex etiology of BMI level and BMI change [19]. As recent GWAS have focused on the BMI level only [63, 64, 104, 130], an interesting perspective would be to perform a GWAS for parameters related to BMI change across life span in order to find novel SNPs robustly associated with more complex obesity phenotypes. The ‘set point’ theory proposes the existence of a feedback biological control system that regulates total body weight to a constant ‘body-inherent’ weight [131]. However, observational studies suggest that a set point in humans, if any, is loose rather than tightly controlled [132] and BMI variance in adulthood is expected to vary significantly in populations, from ‘BMI stable’ to ‘BMI yo-yo’ longitudinal patterns. In line with a growing interest for the genetic analysis on the variance of quantitative traits [133], it may be interesting to estimate the heritability of BMI variance across time and, if substantial, to identify gene variants associated with BMI variance by GWAS. 

Recent GWAS meta-analyses for BMI [63, 64, 104, 130] have collected BMI information in individuals living in a wide range of heterogeneous environments. This approach has been successful but gene variants associated with BMI in specific environmental exposures may have been missed. Knowing that the inter-individual response for BMI distribution to a similar environment can differ considerably and genetic factors must explain these differences in populations [16], GWAS for BMI in specific environmental conditions may lead to the discovery of novel predisposing loci. It may be of particular interest to perform GWAS for BMI dynamic change in response to a brutal obesity-prone environment modification. From this point of view, the genetic analysis of BMI response to antipsychotic drugs intake, pregnancy or smoking cessation would be relevant, as 1) large inter-individual variability, likely to be modulated by genetic factors, is observed for BMI variation in response to these specific environmental exposures and 2) these conditions predispose to subsequent obesity development. GWAS for the BMI response to lifestyle intervention, pharmacotherapy and bariatric surgery may also be useful to identify the genes involved in efficient responses to specific treatments. 

#### On the Use of ‘Deep Phenotyping’ in GWAS

Beyond the study of BMI, GWAS for additional phenotypes such as waist circumference or waist to hip ratio led to the identification of 14 novel genetic loci [110-113]. These data strongly argue for the use of different measures of adiposity in GWAS. Although widely used as “the obesity phenotype” in gene-search studies, BMI is not a good proxy of body fat content, especially at the individual level [134]. Whereas BMI is significantly associated with fat mass in obese subjects, there is little or no association between BMI and fat mass in normal weight and underweight subjects. At a given BMI, fat mass may vary by more than 100% [132]. Because phenotypic precision is major for genetic interrogation, the use of "deep phenotyping" information may help to dissect the genetic influences of obesity [135]. GWAS for endophenotypes such as body fat content and percentage of fat mass estimated by dual-emission X-ray absorptiometry, behavioral food intake measured by *ad libitum* meal test [136] and energy expenditure estimated by room respiration calorimetry [136] may provide highly relevant information. 

Müller *et al*. [137] considered that the genetic basis of body weight regulation is unlikely to be fully discernable in individuals who are at stable energy equilibrium (i.e., at a stable body weight). They proposed to use dynamic phenotypes, including energy intake, energy expenditure and partitioning under conditions of controlled over and underfeeding rather than static phenotypes such as body composition and energy homeostasis. Such dynamic phenotypes should provide a more sensible basis for future genetic studies on obesity [132].

In conclusion, nine loci have been identified in Mendelian forms of non-syndromic obesity, and 58 loci have been robustly associated with polygenic obesity up to now but these loci explain a small fraction of the heritability for obesity. It seems that we are only at ‘the end of the beginning’ of the search for genetic variants that predispose to obesity. Innovative methodologies and technologies presented in detail here lead to optimism, and there is no doubt that we will live a prolific period of discovery in human obesity genetics in the upcoming years. The time required to shift from scientific discoveries to clinical applications is often underestimated [138] but on the longer term, the exhaustive genetic dissection of obesity will help to characterize the disease mechanisms, to provide new targets for drug design, and to lead to an efficient diagnosis, treatment, and prevention of obesity.

## Figures and Tables

**Table 1.** Sixty-One Common Gene Variants at 58 Loci are Associated with Obesity Phenotypes at a Genome-Wide Level of Significance (P < 5 x 10^8^)

Nearest Gene	Polymorphism	Phenotype	Reference
*FTO*	rs1421085 / rs9939609	body mass index, extreme obesity	[99, 100]
*MC4R*	rs17782313	body mass index, extreme and childhood obesity	[107, 109, 130]
*PCSK1*	rs6232	extreme obesity	[74]
*PCSK1*	rs6234 / rs6235	extreme obesity	[74]
*CTNNBL1*	rs6013029	body mass index	[139]
*TMEM18*	rs6548238	body mass index, childhood obesity	[64, 109]
*GNPDA2*	rs10938397	body mass index	[64]
*SH2B1*	rs7498665	body mass index	[64]
*KCTD15*	rs11084753	body mass index	[64]
*MTCH2*	rs10838738	body mass index	[64]
*NEGR1*	rs2815752	body mass index	[64]
*NPC1*	rs1805081	extreme obesity	[107]
*MAF*	rs1424233	extreme obesity	[107]
*PTER*	rs10598503	extreme obesity	[107]
*PRL*	rs4712652	extreme obesity	[107]
*SEC16B*	rs10913469	body mass index	[63]
*ETV5*	rs7647305	body mass index	[63]
*AIF1*	rs2844479	body mass index	[63]
*BDNF*	rs6265	body mass index	[63]
*BDNF*	rs925946	body mass index	[63]
*FAIM2*	rs7138803	body mass index	[63]
*FTO*	rs6499640	body mass index	[63]
*SDCCAG8*	rs12145833	childhood obesity	[109]
*TNKS*	rs17150703	childhood obesity	[109]
*TFAP2B*	rs987237	waist circumference, body mass index	[104, 110]
*MSRA*	rs7826222	waist circumference	[110]
*LYPLAL1*	rs4846567	waist to hip ratio	[110, 113]
*NRXN3*	rs10146997	waist circumference, body mass index	[104, 112]
*C12orf51*	rs2074356	waist to hip ratio	[103]
*GPRC5BB*	rs12444979	body mass index	[104]
*POMC*	rs713586	body mass index	[104]
*MAP2K5*	rs2241423	body mass index	[104]
*GIPR*	rs2287019	body mass index	[104]
*FANCL*	rs887912	body mass index	[104]
*TNNI3K*	rs1514175	body mass index	[104]
*LRRN6C*	rs10968576	body mass index	[104]
*FLJ35779*	rs2112347	body mass index	[104]
*SLC39A8*	rs13107325	body mass index	[104]
*TMEM160*	rs3810291	body mass index	[104]
*CADM2*	rs13078807	body mass index	[104]
*LRP1B*	rs2890652	body mass index	[104]
*PRKD1*	rs11847697	body mass index	[104]
*MTIF3*	rs4771122	body mass index	[104]
*ZNF608*	rs48361333	body mass index	[104]
*PTBP2*	rs1555543	body mass index	[104]
*TUB*	rs4929949	body mass index	[104]
*HMGA1*	rs206936	body mass index	[104]
*MC4R*	rs7227255	body mass index	[104]
*RSPO3*	rs9491696	waist to hip ratio	[113]
*VEGFA*	rs6905288	waist to hip ratio	[113]
*TBX15/WARS2*	rs984222	waist to hip ratio	[113]
*NFE2L3*	rs1055144	waist to hip ratio	[113]
*GRB14*	rs10195252	waist to hip ratio	[113]
*DNM3/PIGC*	rs1011731	waist to hip ratio	[113]
*ITPR2/SSPN*	rs718314	waist to hip ratio	[113]
*LY86*	rs1294421	waist to hip ratio	[113]
*HOXC13*	rs1443512	waist to hip ratio	[113]
*ADAMTS9*	rs6795735	waist to hip ratio	[113]
*ZNRF3/KREMEN1*	rs4823006	waist to hip ratio	[113]
*NISCH/STAB1*	rs6784615	waist to hip ratio	[113]
*CPEB4*	rs6861681	waist to hip ratio	[113]
